# Keys to improving the informed consent process in research: Highlights of the i‐CONSENT project

**DOI:** 10.1111/hex.13427

**Published:** 2022-07-27

**Authors:** Jaime Fons‐Martinez, Cristina Ferrer‐Albero, Javier Diez‐Domingo

**Affiliations:** ^1^ Foundation for the Promotion of Health and Biomedical Research of Valencia Region, FISABIO Valencia Spain; ^2^ Facultad de Medicina y Ciencias de la Salud Universidad Católica de Valencia San Vicente Mártir Valencia Spain

**Keywords:** bioethics, clinical research, comprehension, informed consent, participant‐centred design, research ethics

The ethical and legal governance of all aspects of informed consent in research is becoming increasingly extensive and complex. Instead of a single directive, informed consent is governed by a series of international rules applied to biomedical research, clinical trials and biobanks, while various ethical guidelines for research have been published by different international bodies.

Informed consent is an essential part of any research involving humans, but the array of available guidelines can complicate the informed consent process for sponsors, researchers and participants. Sponsors, in particular, find it difficult to adapt the informed consent process to the characteristics of the participants. Moreover, because of the length and complexity of informed consents, some participants may misconstrue key points^1^ and agree to participate in a trial that they do not fully understand. In these cases, the decision on their participation is mainly based on discussions with the researcher, which lacks traceability.

In 2017, the European Commission responded to the need to improve the informed consent process and informed consent readability by launching the project ‘Improving the guidelines of informed consent, including vulnerable populations, under a gender perspective (i‐CONSENT)’ (Grant Agreement 741856).

The ethical and legal framework of the i‐CONSENT project was later supplemented with the publication *‘Guidelines for Tailoring the Informed Consent Process in Clinical Studies*’, which includes more specific guidelines for developing evidence‐based patient information materials that take into consideration gender, multiculturalism and the vulnerable populations that are usually underrepresented in research. The guidelines also provide a series of easy‐to‐read and easy‐to‐use fact sheets and tools that complement the main document, highlight the importance of various aspects of the informed consent process and offer recommendations on how to implement best practices. These fact sheets include, among others, how to present study information in consent materials; how to assess participant understanding; how to establish an appropriate relationship between the investigator and the participant during the process; and how to address some of the major ethical challenges that may arise in pandemic situations such as COVID‐19.

This article summarizes the key aspects of the informed consent process from the perspective of the i‐CONSENT project.

During the development of the guidelines, multiple reviews of the scientific literature and ethical and legal texts were carried out, as well as workshops, seminars and surveys that allowed us to obtain the opinions on different aspects of informed consent of different people, including representatives of patients and potential participants in clinical studies, experts in legislation, experts in ethics, members of ethics committees, investigators, members of the pharmaceutical industry, legislators and cultural mediators.

The above‐mentioned guidelines and the rest of the project deliverables can be accessed from the CORDIS platform.^2^

## 1 INFORMED CONSENT AS A PROCESS

**Figure 1:**
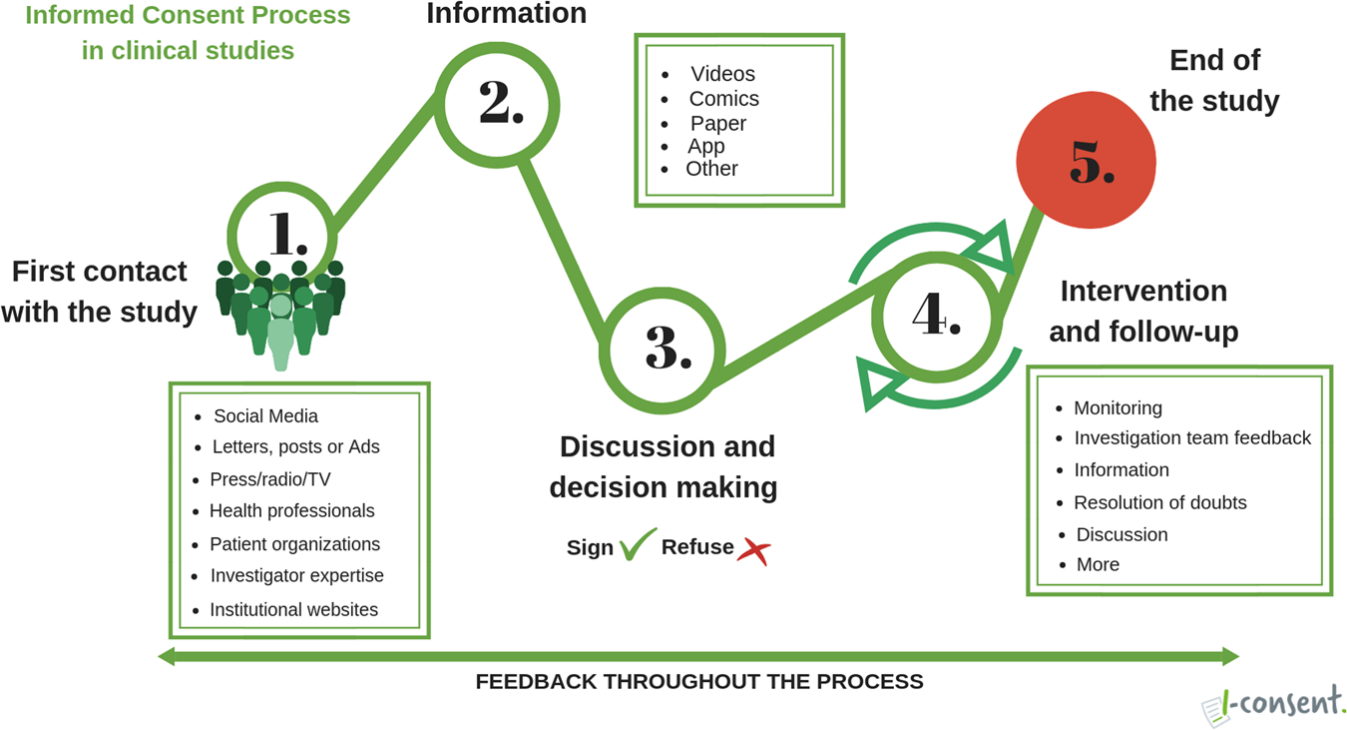
The informed consent process in clinical studies

The main paradigm—an approach suggested earlier by the Council for International Organizations of Medical Sciences—is to view informed consent as a process rather than a bureaucratic procedure aimed merely at obtaining a signature on a document. This guideline identifies and describes five informed consent process phases that are set in motion the moment a potential participant receives information about a particular study and end when the study is completed (Figure 1). It also guides the researcher through each phase of the informed consent process and ensures the autonomy of the potential participant in each phase.

The guidelines, which supplement existing informed consent documentation, introduce novel recommendations in three directions: the adaptation of the informed consent process to potential participants; the improvement of the participant's experience; and the use of new tools to guide the informed consent process. The perspective of potential participants in clinical research was taken into account in the development and design of the guidelines.^2^

## 2 ADAPTATION OF THE INFORMED CONSENT PROCESS TO POTENTIAL PARTICIPANTS

The first recommendation is to adapt the informed consent process to the preferences, interests and needs of the potential participant, focusing on the target population throughout the research process. Representatives of the target population should be involved in all steps, including designing and cocreating the document, implementing the informed consent process and receiving subsequent feedback that can improve the initial process. Design Thinking methodology is recommended to tailor the information to the audience.

This new approach involves two‐way, seamless interaction with participants^3^ that allows the researcher to detect and clarify concepts that are likely to be misunderstood, especially by people with low health literacy, and avoids overwhelming potential participants with excessive information. The strategy of providing information in layers allows participants to decide for themselves how much information they receive about a research study.

## 3 PRESENTING INFORMATION IN DIFFERENT FORMATS

In today's world, reading and learning habits have changed, and written texts now include other elements such as hyperlinks, multimedia, images and infographics. The informed consent should be tailored to social changes that facilitate understanding and should be presented in different formats, which may or may not be combined with new technologies.^4^ The participants, depending on their personal characteristics, may choose the format that best suits their preferences and needs.

## 4 NEW TOOLS TO IMPROVE COMMUNICATION

The guidelines include practical tools and checklists that help users meet regulatory and stakeholder requirements and identify and review all key aspects that must be covered by the informed consent process. This approach will improve understanding and satisfy the needs and preferences of potential participants.

The guidelines also include 14 fact sheets and six tools that highlight the different issues addressed in the informed consent process and offer recommendations on how to implement best practices. The fact sheets explore in greater depth topics such as presenting the informed consent, evaluating comprehension, information and using decision‐making tools. The tools address matters that are not strictly related to the informed consent process, but that are useful for improving the process, for example, communication skills, writing a thank you letter or methods for incorporating the perspective of the participants.

## 5 GUIDELINES' VALIDATION

The recommendations put forward have been validated at several levels.

The RAND/UCLA method for validating clinical guidelines was used to analyse and validate the appropriateness of the main recommendations, particularly the most innovative aspects.^5^ The evaluation panel comprised patient representatives, investigators, experts in ethics, pharmaceutical industry representatives and regulators, all of them external to the project. Fifty‐three recommendations were evaluated. Of these, 43 were considered ‘appropriate’; 10 were considered ‘uncertain’; and none were considered ‘inappropriate’. All recommendations rated medians of 6.5–9 on a 1–9 scale (1 = ‘extremely inappropriate’, 5 = ‘uncertain’, 9 = ‘extremely appropriate’). Discrepancies were discussed by the expert panel, and some recommendations were adapted.

To validate the recommendations in a target population, three pilot consent forms were designed for hypothetical clinical trials with vaccines, one for children, one for pregnant women and one for adults, in three culturally different countries. Since these were not real clinical trials, only the recommendations for drafting information (step 2 of the Informed Consent Process; see Figure 1) were taken into consideration in the informed consent process. These recommendations include the involvement of potential participants in the design and piloting of consent materials. In two of the three hypothetical clinical trials, materials were cocreated with potential participants through design thinking sessions. In the third, a survey was conducted to learn the needs and preferences of potential participants. All three materials were piloted with potential participants.

To finalize the project, the guidelines were used to design patient information for the VIGIRA study (EudraCT No. 2019‐001186‐33, funded by Instituto de Salud Carlos III Research Grants) on the effects of an influenza vaccine in children aged 12–35 months during the 2019–2020 and 2020–2021 influenza seasons. The materials were designed WITH and FOR parents of children who could potentially participate in the study. In this case, cocreation was done through interviews with parents of potential participants. In addition, feedback from researchers and participants of the study in previous seasons was also used.

The i‐CONSENT project has compiled and analysed legislation and ethical recommendations applicable in Europe, identifying the aspects that generate most uncertainty for the investigator, for example: how to adapt it to the needs of the potential participant, how to express it in plain language, how to assess its comprehension, how to apply gender and multicultural perspectives, and so forth. This analysis has made possible the elaboration of more specific recommendations on the informed consent process, which help to achieve the objectives set by the international bodies responsible for guaranteeing the protection and autonomy of patients participating in medical research.

The recommendations of the i‐CONSENT project have been developed to complement and facilitate the implementation of international ethical guidelines and European and national legislation on clinical research.

## Data Availability

The article has no data.
